# Epidemiology of hepatitis E virus infection in animals in Africa: a systematic review and meta-analysis protocol

**DOI:** 10.1186/s13643-019-1038-0

**Published:** 2019-05-20

**Authors:** Abdou Fatawou Modiyinji, Jean Joel Bigna, Fredy Brice N. Simo, Moise Nola, Marie S. Ndangang, Sebastien Kenmoe, Richard Njouom

**Affiliations:** 1 Department of Virology, Centre Pasteur of Cameroon, 451 Rue 2005, P.O. Box 1274, Yaoundé, Cameroon; 2 Department of Biology and Animal Physiology, Faculty of Sciences, University of Yaoundé I, P.O. Box 337, Yaoundé, Cameroon; 3 Department of Epidemiology and Public Health, Centre Pasteur of Cameroon, 451 Rue 2005, P.O. Box 1274, Yaoundé, Cameroon; 4 School of Public Health, Faculty of Medicine, University of Paris Sud, 63 Rue Gabriel Péri, 94270 Le Kremlin-Bicêtre, France; 5 Department of Biochemistry, Faculty of Sciences, University of Yaoundé I, P.O. Box 337, Yaoundé, Cameroon; 6 Department of Medical Information and Informatics, Rouen University Hospital, Rouen, France

**Keywords:** Hepatitis E, Africa, Epidemiology, Animals

## Abstract

**Background:**

Hepatitis E virus (HEV) is one of the major causes of acute hepatitis in humans worldwide with a case-fatality rate of 1–4% in the general population that might reach 30% in pregnant women. In the past decade in Africa, studies have shown that HEV infects not only humans but also animals. A systematic review summarizing the epidemiological data of HEV has been only performed in humans residing in Africa. We will perform this systematic review and meta-analysis to assess the prevalence of HEV infection in animal species in Africa.

**Methods:**

This review will include cross-sectional studies among different animal species that reported the prevalence of HEV in Africa. We will consider published and unpublished studies from January 1, 2000 to present. A comprehensive search of PubMed, Excerpta Medica, African Journals Online, and Africa Index Medicus will be conducted to identify all relevant articles. Reference lists of eligible items and relevant articles will be reviewed. The funnel plots and the Egger test will be used to assess the publication bias. Study-specific estimates will be aggregated using a DerSimonian and Laird random-effects meta-analysis model to obtain an overall summary estimate of HEV prevalence across studies. The heterogeneity of the studies will be evaluated by the *χ2* test on the Cochran’s *Q* test. The results will be presented by animal species.

**Discussion:**

HEV-infected animals are likely to transmit this virus to humans in Africa, as studies have already shown in developed countries. This systematic review and meta-analysis will provide a clear picture of the epidemiology of HEV in animals in Africa, to better understand this infection and to respond adequately to the epidemic challenges that often afflict Africa.

**Systematic review registration:**

PROSPERO, CRD42018087684.

**Electronic supplementary material:**

The online version of this article (10.1186/s13643-019-1038-0) contains supplementary material, which is available to authorized users.

## Background

Hepatitis E virus (HEV) is an important cause of acute hepatitis in humans worldwide with a case-fatality rate of 1–4% in the general population that might reach 30% in pregnant women [1]. HEV is responsible for 20–50% of acute hepatitis in developing countries [1]. In 2010, 20 million people were infected with HEV [2]. This virus infects not only humans but also animals. HEV belongs to the family of Hepeviridae and the genus Hepevirus containing four species, three infecting mammals (Orthohepevirus A, C, and D) and one infecting birds (Orthohepevirus B). Only Orthohepevirus A contains strains that are transmissible to humans and animals. The genus Orthohepevirus A is currently classified into seven genotypes (HEV-1 to HEV-7) [3]. The epidemiology of these genotypes depends on the geographical region. In industrialized countries, genotypes 3 and 4, which cause sporadic cases, can be transmitted to humans from infected animals [4]. On the other hand, genotypes 1 and 2, through contaminated water, are responsible for major epidemics in developing countries [5]. To date, oral-fecal transmission is the only route of infection documented in Africa. Nevertheless, other sources of infection such as zoonotic transmission cannot be ruled out in Africa. Indeed, genotype 3, responsible for the zoonotic transmission of HEV in developed countries is already present in some African studies conducted in humans [6, 7]. In addition, several studies in Africa have shown that people who have regular contact with animals such as slaughterers, pig farmers, or veterinarians have a higher prevalence of anti-HEV antibodies than the general population [8]. From these studies, it is clear that animals play an important role in the epidemiology of HEV in Africa.

To date, there is only one systematic review providing epidemiological data on hepatitis E virus in humans in Africa, but data in animals are still disparate [8]. Indeed, to the best of our knowledge, no study giving a global idea of the epidemiology of HEV in animals in Africa is yet available. In order to fill this gap, this review will be an opportunity to discuss the epidemiological data of HEV in animal species and reservoirs of HEV. The objective of this systematic review and meta-analysis is therefore to provide a summary of data on the prevalence and different genotypes of HEV in animals in Africa.

## Methods/design

This protocol was reported in accordance with PRISMA for protocol guidelines (see Additional file 1).

### Eligibility criteria

#### Inclusion criteria

1. Population: studies carried out in all animal species living in Africa;
2. Types of studies: cross-sectional studies;
3. Types of diagnostic: studies that report the seroprevalence of anti-HEV antibodies (immunoglobulins G, immunoglobulins M) and/or the prevalence of viral ribonucleic acid;
4. Types of publications: published and unpublished studies, reported from January1, 2000 to present;
5. No language restrictions will be applied.

#### Exclusion criteria

1. Studies that report imported cases of HEV infections in animal in Africa;
2. Studies in human participants;
3. Studies with experimental infections of animals in laboratory;
4. In case of duplicate reports, the most complete and up-to-date version will be considered for this review;
5. Studies whose prevalence data will not be accessible even after the request to the authors.

### Strategy to identify relevant studies

The search strategy will be implemented in two steps:

#### Bibliographic database searches

A comprehensive search of MEDLINE through PubMed, Excerpta Medica, African Journals Online, and Africa Index Medicus will be conducted to identify all relevant articles published on HEV in animals living in Africa from January 1, 2000 to present, without any linguistic restrictions. A search strategy based on the combination of relevant terms will be designed and applied. Text words and medical subject header terms will be used. The following terms and their variants will be used for hepatitis E virus: “HEV,” “Hepatitis E,” and “Hepatitis E.” Individual country names for the 54 African countries and names of African sub-regions such as “West Africa” or “East Africa” will also be used as additional key search terms for more abstracts on the subject. The names of African countries will be introduced in both English and the languages relevant to each country, for example, “Ivory Coast” and “Côte d’Ivoire.” When country names have changed over time, old, and new names will be included, such as “Zaire” and “Democratic Republic of Congo”. Abstracts of all eligible articles will be reviewed and full texts of articles will be available. The main search strategy in PubMed/MEDLINE is shown in Table 1. This search strategy will be adapted for search in other databases. A filter will be applied to select studies in animals. We have built the search strategy for electronic databases as per PRESS (Peer Review of Electronic Search Strategies) guidelines [9].

**Table 1:** Search strategy in PubMed

Search	Search terms
#1	“Hepatitis E virus” OR “HEV” OR “Viral hepatitis E”
#2	(Africa OR Algeria OR Angola OR Benin OR Botswana OR “Burkina Faso” OR Burundi OR Cameroon OR “Canary Islands” OR “Cape Verde” OR “Central African Republic” OR Chad OR Comoros OR Congo OR “Democratic Republic of Congo” OR Djibouti OR Egypt OR “Equatorial Guinea” OR Eritrea OR Ethiopia OR Gabon OR Gambia OR Ghana OR Guinea OR “Guinea Bissau” OR “Ivory Coast” OR “Cote d’Ivoire” OR Jamahiriya OR Kenya OR Lesotho OR Liberia OR Libya OR Madagascar OR Malawi OR Mali OR Mauritania OR Mauritius OR Mayotte OR Morocco OR Mozambique OR Namibia OR Niger OR Nigeria OR Principe OR Reunion OR Rwanda OR “Sao Tome” OR Senegal OR Seychelles OR “Sierra Leone” OR Somalia OR “South Africa” OR “St Helena” OR Sudan OR Swaziland OR Tanzania OR Togo OR Tunisia OR Uganda OR “Western Sahara” OR Zaire OR Zambia OR Zimbabwe OR “Africa” OR “African”) NOT (“guinea pig” OR “guinea pigs” OR “aspergillus niger”)
#3	# 1 AND # 2
#4	#3 Limits: From January 1, 2000 to present and animals studies

#### Searching for the other sources

A manual search that consists of scanning reference lists of eligible items and other relevant articles will be performed.

### Selection of studies to include in the review

The selection of studies will be managed using EndNote X7. Two researchers will independently identify the articles and sequentially review their titles and/or abstracts to determine their eligibility. The full text of articles potentially eligible will be acquired. These researchers will independently assess the full text of each study to determine eligibility, and in a consensual way, select which studies to include. Existing disagreements will be resolved by a third author. We will use a selection guide to ensure that the selection criteria are reliably applied by all evaluators. Eligible studies in languages other than English, French, or Spanish will be translated using Google Translate and considered for inclusion.

### Assessment of methodological quality and reporting of data

An adapted version of the risk of bias tool for prevalence studies developed by Hoy et al. [10] will be used to evaluate included studies for methodological quality and risk of bias. The defined items will be scored with 0 for ‘no’ and 1 for ‘yes.’ The total score of each article will be calculated by the sum of its items. The studies will be ranked according to their total score as low risk, moderate risk, and high risk for scores of 8–10, 5–7, and 0–4, respectively. Two review authors will assess the risk of bias and disagreements will be solved through a consensus or by an arbitration of a third review author.

### Data extraction and management

Two researchers will extract data concerning:

1. Details of the author: name of first author and year of publication;
2. Characteristics of the study: recruitment site (country, city), setting (rural and/or urban), sampling method (random, systematic);
3. Characteristics of participants: species, number of animals examined, number of animals infected with hepatitis E virus, age distribution, proportion of male animals, criteria for inclusion, and exclusion of animals.

When only primary data (number of animals examined and number of animals infected with hepatitis E) are provided, these data will be used to calculate prevalence estimates. The data will be extracted using a preconceived, piloted, and standardized data abstraction form. In case of missing data, we will contact directly the corresponding author to request the information. In the case of multinational studies, the results will be separated to show the estimate in each country. When it is not possible to disaggregate the data by country, the study will be presented in one and the countries in which the study was conducted will be presented.

### Data synthesis including assessment of heterogeneity

The data will be analyzed using the “meta” packages of the R statistical software (version 3.3.3, The R Foundation for statistical computing, Vienna, Austria). The prevalence of HEV infection will be recalculated on the basis of numerators and denominators provided by individual studies. To minimize the effect of studies with extremely small or extremely large prevalence estimates on the overall estimate, the variance in study-specific prevalence will be stabilized with Freeman-Tukey arc-sinetransformation before pooling the data with the random effects meta-analysis model [11]. Heterogeneity will be assessed by the chi-square test on Cochran’s *Q* test [12], which will be quantified by *I*^2^ values, assuming *I*^2^ values of 25, 50, and 75% respectively representative low, medium, and high heterogeneity [13]. When substantial heterogeneity is detected, we will perform meta-regression and subgroup analyses to investigate possible sources of heterogeneity using the following grouping variables: mean or median age, gender, study setting (rural, urban), geographical area (north Africa, central, western, eastern, southern), sampling method, and country. The symmetry of the funnel plots and the Egger test will be performed to assess the presence of publication bias and selective reporting [14]. A *p* value < 0.10 will be considered indicative of a statistically significant publication bias. Subgroup analyses will be conducted according to the animal species.

### Reporting and presentation of results

The resulting systematic review and meta-analysis will follow the meta-analysis of Observational Studies in Epidemiology guidelines for reporting [15]. The study selection process will be summarized using a flow diagram including reasons for exclusion of the studies. The summary tables and forest plots will be used to summarize the quantitative data.

## Discussion

HEV-infected animals are likely to transmit this virus to humans in Africa, as studies have already shown in developed countries [4]. This systematic review and meta-analysis should provide a clear picture of the epidemiology of HEV in animals in Africa, to better understand this infection and to respond adequately to the epidemic challenges that often afflict Africa. For this review, we recognized that there may be strong heterogeneity in estimating prevalence due to the diversity of animal species studied. This review does not require ethical approval because it is based on published studies. The final report of the systematic review, in the form of a scientific paper, will be published in a peer-reviewed journal. In addition, the results will be presented at conferences and submitted to the relevant health authorities.

## Additional file

Additional file 1: PRISMA-P (Preferred Reporting Items for Systematic review and Meta-Analysis Protocols) 2015 checklist: recommended items to address in a systematic review protocol*. (PDF 225 kb)

## Abbreviations

HEV: Hepatitis E virus

## Authors’ information

This study is a part of the PhD thesis of AFM.
